# A multidimensional assessment of adverse events associated with paliperidone palmitate: a real-world pharmacovigilance study using the FAERS and JADER databases

**DOI:** 10.1186/s12888-025-06493-0

**Published:** 2025-01-20

**Authors:** Siyu Lou, Zhiwei Cui, Yingyong Ou, Junyou Chen, Linmei Zhou, Ruizhen Zhao, Chengyu Zhu, Li Wang, Zhu Wu, Fan Zou

**Affiliations:** 1 Department of Respiratory and Critical Care Medicine, Affiliated Hospital of Zunyi Medical University, 149 Dalian Road, huichuan district, Zunyi, Guizhou 563003 People’s Republic of China; 2 Department of Obstetrics and Gynecology, The First Affiliated Hospital of Xi’an Jiaotong University, Xi’an, China

**Keywords:** Paliperidone palmitate, FAERS, JADER, Adverse drug events, Disproportionality analysis, Real-world analysis

## Abstract

**Objective:**

Paliperidone palmitate is a second-generation antipsychotic that has undergone extensive investigation in clinical trials. However, real-world studies assessing its safety in large populations are lacking. As such, this study aimed to comprehensively evaluate real-world adverse drug events (ADEs) linked to paliperidone palmitate by employing data mining techniques on the U.S. Food and Drug Administration Adverse Event Reporting System (FAERS) database and the Japanese Adverse Drug Event Report (JADER) database.

**Methods:**

The study retrieved ADE reports from the FAERS database covering the period from 2009 through the third quarter of 2024, and from the JADER database covering the period from 2013 through the second quarter of 2024. Utilizing disproportionality analyses such as the reporting odds ratios (ROR), proportional reporting ratios (PRR), Bayesian confidence propagation neural network (BCPNN), and multi-item Poisson shrinkage (MGPS), significant associations between ADEs and paliperidone palmitate were evaluated.

**Results:**

A total of 27,672 ADE reports related to paliperidone palmitate were identified in FAERS, with 285 significantly disproportionate preferred terms (PTs) identified by all four algorithms. Paliperidone palmitate-associated ADEs encompassed 27 System Organ Classes (SOCs). The top three PTs with the highest reported cases were off-label use, drug ineffective, and hospitalization. Common ADEs included increased blood prolactin, galactorrhea, and schizophrenia, which was consistent with drug label. Noteworthy, unexpected signals not listed in the drug label were also identified, such as psychosexual disorders, prolactin-producing pituitary tumors, suicide attempt, and sudden death. The median onset time for all ADEs was 40 days. Furthermore, gender-based difference in risk signals was detected. Females are more likely to experience elevated blood prolactin and weight increase, whereas males are more prone to sexual dysfunction. Among the 1,065 ADE reports from the JADER database, we identified 51 positive signals, 35 of which overlapped with those found in FAERS, including schizophrenia, hyperprolactinemia, and erectile dysfunction.

**Conclusion:**

The study findings from two independent databases serve as crucial references for ensuring the safe of paliperidone palmitate. Additionally, the gender-specific monitoring references provided can enhance clinical surveillance efforts and facilitate more effective risk identification.

**Supplementary Information:**

The online version contains supplementary material available at 10.1186/s12888-025-06493-0.

## Background

 Schizophrenia and schizoaffective disorder are grave chronic mental conditions that substantially impact life expectancy and functional capabilities [26, 69]. These disorders are linked to severe disability, hampering both academic and occupational performance. Schizophrenia, in isolation, accounts for 15.1 million years of life with disability (YLDs) in the global burden of disease [64]. The primary treatment for patients with schizophrenia revolves around antipsychotic medications, essential for managing acute psychotic episodes and preventing relapses of the disorder [29, 54].

Paliperidone, a second-generation antipsychotic, received approval for the treatment of schizophrenia in 2006 and schizoaffective disorder in 2009. Paliperidone palmitate, the palmitate ester of paliperidone and the primary metabolite of risperidone, is formulated as a long-acting injection (LAI) for intramuscular administration. It shares a similar pharmacology to risperidone, acting as an antagonist at D2 and 5-HT(2 A) receptors [6]. LAI formulations enhance treatment adherence, with paliperidone available in multiple long-acting forms such as monthly, tri-monthly, and six-monthly injections [13, 74]. These LAIs offer advantages over oral antipsychotics by bypassing liver and intestinal absorption, directly entering the circulatory system to reduce the “first-pass effect,” enhance bioavailability, and minimize drug-related side effects [73]. Moving forward, paliperidone palmitate holds promise for diverse applications, warranting further investigation into its efficacy and tolerability.

Drug safety has always been a prominent concern in drug development and clinical practice [77]. Striking a balance between a drug’s effectiveness and safety is paramount, especially in the treatment of mental health conditions. The FDA Adverse Event Reporting System (FAERS) database stands out as a vital information source for real-world adverse drug events (ADEs)(Cui et al., [17]. This extensive database encompasses millions of adverse event reports submitted by medical professionals, pharmacists, manufacturers, and others. It stands as the largest and most comprehensive post-marketing safety surveillance database globally. FAERS aids researchers, healthcare providers, and regulatory agencies in monitoring drug safety and comprehending the efficacy of medications, particularly concerning severe and uncommon adverse events. The Japanese Adverse Drug Event Report (JADER) database, maintained by the Pharmaceuticals and Medical Devices Agency (PMDA) in Japan, serves as a comprehensive repository for documenting and managing drug-related adverse events reported within the country [53]. This study aimed to comprehensively analyze ADE reports associated with paliperidone palmitate in the FAERS database. The investigation focused on gender-differentiated safety signals, employing a range of signal detection methods to examine these differences from multiple perspectives. Additionally, the study sought to identify unexpected safety signals not listed in the drug label and to evaluate the onset time of these ADEs. To enhance the robustness and reliability of the findings, external validation was conducted using data from the JADER database.

## Methods

### Data sources and pre-processing

We conducted a retrospective pharmacovigilance analysis utilizing the FAERS and JADER databases. FAERS, a publicly available database, compiles all self-reported adverse event accounts documented by the FDA. Presently, the FAERS database holds over 10 million case reports. For this study, all instances of paliperidone palmitate in the FAERS database since FDA approval were retrieved for further examination. Critical details, such as patient demographics (age, gender, weight), reporting country, reporter type, indication, suspected drug, adverse drug events, and patient outcomes, were retrieved and subjected to analysis. The JADER database has been compiling case reports submitted by pharmaceutical companies and medical institutions since April 2004. It consists of four main files: DEMO, DRUG, REAC, and HIST [30]. The “DEMO” file provides fundamental patient information, including gender, age, and weight. The “DRUG” file contains details about the drug, such as its generic name, the route of administration, and the start and end dates of treatment. The “REAC” file documents the adverse event’s name, the outcome, and the date of occurrence. Lastly, the “HIST” file includes information about the patient’s underlying medical conditions [31].

**Fig. 1:**
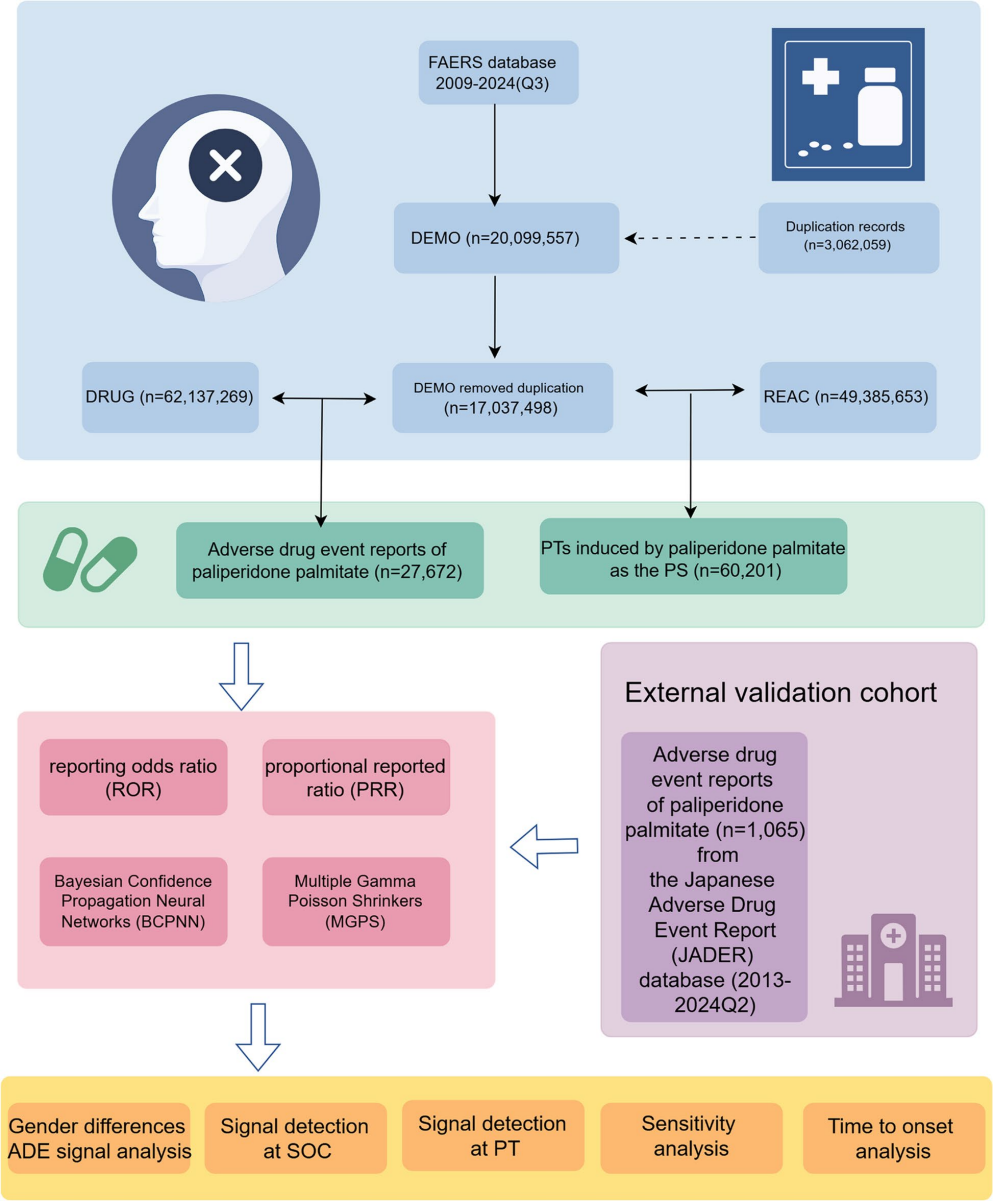
Flowchart of the whole study. FAERS, Food and Drug Administration Adverse Event Reporting System; PTs, preferred terms; PS, primary suspect; ADE, adverse drug event. Q3, third quarter; Q2, second quarter

To amass reports of ADEs linked to the administration of paliperidone palmitate, we conducted searches from January 1, 2009 (Q1 2009) to September 30, 2024 (Q3 2024). Due to the lack of a standardized drug coding system in the FAERS database, searches were performed using both generic and brand names, including “INVEGA SUSTENNA,” “PALIPERIDONE PALMITATE,” and “INVEGA TRINZA.” Since the FAERS database is updated quarterly and may include duplicate reports or entries that have been withdrawn or deleted, we implemented a deduplication process in accordance with FDA’s recommended criteria: selecting the most recent FDA_DT when CASEIDs were identical, and opting for the higher PRIMARYID in cases of identical CASEIDs and FDA_DTs [17]. Through the de-duplication process, a total of 3,062,059 duplicate reports were successfully identified and removed. ADEs were categorized based on the top-level classification of the Medical Dictionary for Regulatory Activities (MedDRA, version 27.0), employing System Organ Class (SOC) terms [10]. Preferred terms (PTs) are the foundational terminology in MedDRA, offering standardized and accurate descriptions of medical events and concepts. Moreover, in a bid to enhance result accuracy and mitigate potential impacts from concurrent medications, we maintained role codes solely for adverse events attributed to the primary suspect (PS) medications. Using the aforementioned method, we retrieved a total of 27,672 ADE reports related to paliperidone palmitate from the FAERS database. Additionally, 60,201 paliperidone palmitate-associated PTs were identified as PS. In the JADER database, we analyzed data spanning the period from January 1, 2013 to June 30, 2024. To ensure data accuracy, duplicate reports were removed from the DRUG and REAC files. The DEMO table was then linked to these files based on predefined conditions [33]. “パリペリドンパルミチン酸エステル” was used for retrieval. The drugs were divided into three categories—PS, concomitant drugs (C), and drug interactions (I)—based on their influence on adverse events. Following the same methodology used in FAERS, the role code was designated as PS. MedDRA version 27.0 was used to encode Japanese medical terms and their English translations in JADER [79]. This helped to reduce bias in data analysis due to differences in database formats and ensured that ADE terminology was comparable between the two databases [79]. Through the above steps, a total of 1,065 ADE reports associated with paliperidone palmitate were collected in JADER (Fig. 1).

### Data analysis

The reporting odds ratios (ROR), proportional reporting ratios (PRR)(Evans et al., [20], Bayesian confidence propagation neural network (BCPNN)(Bate et al., [4], and multi-item gamma Poisson shrinker (MGPS)(Kubota et al., [38] methods within the disproportionality methods were utilized to detect signal intensity. By employing multiple algorithms simultaneously, we optimally leveraged the advantages of different algorithms to broaden the detection range. This approach enabled a comprehensive review of results from various perspectives to identify safety signals that are more robust and reliable. Additionally, the Bayesian-based algorithm we utilize is designed to adapt to varying data distributions and structures, effectively addressing data variability. This ensures the generation of consistent and reliable signals, facilitating the comparability of results across the two databases [1, 21]. All algorithms are based on 2 × 2 contingency tables (as depicted in Table 1). To enhance result reliability, only PTs that concurrently satisfied all four algorithms were considered positive signals. To minimize the risk of false-positive results (type I errors), we used the Bonferroni method to adjust for multiple *P*-value comparisons [18]. The adjusted significance threshold was determined using the formula: Bonferroni-corrected *P*-value = *P*/n, where *P* is the initial significance threshold, and n is the total number of tests performed. ADEs related to drug indications were excluded to enhance statement clarity. Additionally, signals not listed on the drug label were classified as unexpected signals. The study’s flow chart is presented in Fig. 1.

**Table 1:** A two-by-two contingency table and detailed formulas for disproportionality analysis

	**Target adverse drug event**	**Other adverse drug events**	**Sums**
**Paliperidone palmitate**	a	b	a+b
**Other drugs**	c	d	c+d
**Sums**	a+c	b+d	a+b+c+d
Algorithms	**Equation**		**Criteria**
ROR	ROR = ad/bc		lower limit of 95% CI>1, N≥3
	95 % CI = eln(ROR) ± 1.96(1/a+1/b+1/c+1/d)^0.5		
PRR	PRR = [a(c+d)]/[c(a+b)]		PRR≥2, χ2≥4, N≥3
	χ2 = [(ad-bc)^2](a+b+c+d)/[(a+b)(c+d)(a+c)(b+d)]		
BCPNN	IC=log2a(a+b+c+d)/[(a+c)(a+b)]		IC025>0
	95 % CI = E(IC) ± 2[V(IC)]^0.5		
MGPS	EBGM = a(a+b+c+d)/[(a+c)(a+b)]		EBGM05>2
	95 % CI = eln (EBGM) ± 1.96(1/a+1/b+1/c+1/d)^0.5		

The methods, formulas, and thresholds for Reporting Odds Ratio (ROR), Proportional Reporting Ratio (PRR), Bayesian Confidence Propagation Neural Network (BCPNN), and Empirical Bayesian Geometric Mean (EBGM) are outlined. a, the count of reports featuring both the specified drug and target adverse events; b, the number of reports involving other adverse drug events alongside the specified drug; c, the reports of target adverse drug events involving other drugs; d, the reports encompassing other drugs and non-targeted adverse drug events. 95% CI, 95% confidence interval; N, number of reports; χ2, chi-squared; IC, information component; IC025, lower limit of 95% CI of the IC; E(IC), IC expectations; V(IC), variance of IC; EBGM05, lower limit of 95% CI of EBGM

### Time to onset (TTO) analysis

In this study, we defined the time to onset (TTO) for ADEs associated with paliperidone palmitate as the period between the date of ADE occurrence in the DEMO file (EVENT_DT) and the date of dosing initiation reported in the THER file (START_DT). Cases with missing or incomplete dates were excluded, as were cases with inaccuracies in date specification (e.g., lacking a specific day, month, or year). Furthermore, cases where the ADE onset date preceded the initiation date of paliperidone palmitate therapy were omitted, as this would lead to a negative time-to-onset calculation. Furthermore, we excluded outliers and anomalies to enhance the accuracy and reliability of the TTO analysis. By utilizing the Weibull distribution test, we effectively identified and estimated any fluctuations in ADE risk incidence over time [47]. The Weibull distribution test, defined by the scale (α) and shape (β) parameters, can be used to identify and predict changes in the risk incidence of ADEs over time, with the shape parameter β serving as the primary focus in this study. When β is less than 1, and its 95% confidence interval (CI) also falls below 1, the risk of adverse effects is considered to decrease over time, reflecting an early failure-type curve. Conversely, if β is approximately equal to 1 and its 95% CI includes the value 1, the risk is deemed persistent over time, corresponding to a random failure-type curve. Lastly, if β is greater than 1 and its 95% CI excludes the value 1, the hazard is interpreted as increasing over time, indicative of a wear-out failure-type curve [59]. The cumulative incidence of ADEs in patients treated with paliperidone palmitate was illustrated using the Kaplan-Meier method and compared through the log-rank test [34].

### Statistical analysis

All data processing and statistical analyses were conducted utilizing Microsoft Excel 2019 and R software (version 4.2.1). The “ggplot2” package in R software was employed for data visualization.

## Results

### Descriptive characteristics

In this study, following the elimination of duplicates, a total of 27,672 ADE reports and 60,201 paliperidone palmitate-associated PTs were collected from the FAERS database between Q1 2009 and Q3 2024 (Fig. 1). Of all reports submitted (27,672), 45.3% were submitted by males (*n* = 12,530) and 31.0% by females (*n* = 8,572). Age data was available for 10,243 patients, with the highest proportion (33.4%) falling within the 18–65 years bracket. Reports including weight-specific details numbered 4,424, with breakdowns of 0.7%, 12.2%, and 3.1% for < 50 kg, 50–100 kg, and > 100 kg, respectively. The United States accounted for the majority of recorded information (74.9%). Healthcare professionals submitted the majority of reports (*n* = 21,680, 78.3%), enhancing the reliability of the analysis. Regarding clinical outcomes, hospitalization-initial or prolonged (20.3%) had the highest occurrence among serious adverse events, followed by other serious outcomes (17.5%). Schizophrenia was the most frequent indication reported (*n* = 7,271, 26.3%), followed by schizoaffective disorder (*n* = 1,426, 5.2%). Additional details can be found in Table 2.

**Table 2:** Demographic characteristics of ADEs reported in the FAERS database (January 2009- September 2024) with paliperidone palmitate as the primary suspect drug

**Characteristics**	**Case number**	**Case proportion, %**
**Gender, n (%)**	27672	
Female	8572	31.0%
Male	12530	45.3%
Unknown	6570	23.7%
**Age**		
<18 years	239	0.9%
18-65 years	9254	33.4%
>65 years	750	2.7%
Unknown	17429	63.0%
**Weight**		
<50 kg	202	0.7%
50-100 kg	3372	12.2%
>100 kg	850	3.1%
Unknown	23248	84.0%
**Reported Countries (top five)**		
United States	20732	74.9%
France	915	3.3%
Japan	868	3.1%
United Kingdom	754	2.7%
Colombia	591	2.1%
**Reported person**		
Health professionals	21680	78.3%
Consumer	5897	21.4%
Unknown	95	0.3%
**Outcome**		
Hospitalization-initial or prolonged	5618	20.3%
Life-threatening	412	1.5%
Disability	208	0.8%
Congenital anomaly	6	0.0%
Death	1305	4.7%
Other serious outcome	4833	17.5%
Required intervention	6	0.0%
Unknown	15284	55.2%
**Indication (top five)**		
Schizophrenia	7271	26.3%
Schizoaffective disorder	1426	5.2%
Biopolar disorder	705	2.5%
Psychotic disorder	606	2.2%
Schizophrenia; paranoid type	242	0.9%
**Report year**		
2009	22	0.1%
2010	620	2.2%
2011	552	2.0%
2012	1271	4.6%
2013	1866	6.7%
2014	1732	6.3%
2015	1844	6.7%
2016	1831	6.6%
2017	3010	10.9%
2018	2340	8.5%
2019	2173	7.9%
2020	1958	7.1%
2021	1871	6.8%
2022	2666	9.6%
2023	2195	7.9%
2024 (Q3)	1721	6.2%

*ADEs*Adverse drug events, *Kg* Kilogram, *Q3* Third quarter

### Signal detection at the SOC level

The Table S1 delineates the signal intensity and reporting of adverse effects related to paliperidone palmitate at the SOC level. A total of 27 organ systems displayed adverse events associated with paliperidone palmitate. The most prevalent SOCs in terms of ADE occurrences were psychiatric disorders (*n* = 11,690), general disorders, and administration site conditions (*n* = 11,582), and injury, poisoning and procedural complications (*n* = 8,976). Significant SOCs, meeting the criteria of at least one of the four disproportionality analysis methods, included injury, poisoning, and procedural complications (SOC code: 10022117), general disorders, and administration site conditions (SOC code: 10018065), psychiatric disorders (SOC code: 10037175), nervous system disorders (SOC code: 10029205), investigations (SOC code: 10022891), reproductive system and breast disorders (SOC code: 10038604), surgical and medical procedures (SOC code: 10042613), product issues (SOC code: 10077536), social circumstances (SOC code: 10041244), and endocrine disorders (SOC code: 10014698). Remarkably, psychiatric disorders (ROR 4.06, PRR 3.47, EBGM05 3.40, IC025 1.76), reproductive system and breast disorders (ROR 4.57, PRR 4.44, EBGM05 4.26, IC025 2.08), surgical and medical procedures (ROR 2.35, PRR 2.31, EBGM05 2.22, IC025 1.14), and social circumstances (ROR 3.33, PRR 3.30, EBGM05 3.11, IC025 1.62) were the sole SOCs meeting all four criteria concurrently.

### Signal detection at the PT level

Figure S1 presents the top 50 PTs with the highest percentages for paliperidone palmitate, categorized by the number of case reports. The leading three PTs were off label use (4.35%), drug ineffective (2.87%), and hospitalization (2.76%). After excluding PTs that could serve as potential indications for paliperidone palmitate, 285 PTs were identified as significantly disproportionate, meeting all four criteria in the disproportionality analysis methods concurrently (Table S2). These PTs were further ranked based on the outcomes of the rigorous EBGM algorithm, with the top 50 listed in Table 3. Noteworthy findings include blood prolactin (*n* = 3, ROR 222.49, PRR 222.48, IC 7.45, EBGM 175.02), blood prolactin increased (*n* = 802, ROR 216.82, PRR 213.94, IC 7.41, EBGM 169.70), blood prolactin abnormal (*n* = 82, ROR 93.42, PRR 93.29, IC 6.39, EBGM 83.62), galactorrhea (*n* = 531, ROR 84.07, PRR83.33, IC 6.24, EBGM 75.70), and schizophrenia (*n* = 837, ROR 74.85, PRR 73.82, IC 6.08, EBGM 67.78), which align with the drug’s label. Additionally, unexpected signals denoted by an asterisk were identified, such as psychosexual disorder (*n* = 36, ROR 132.96, PRR 132.88, IC 6.84, EBGM 114.41), prolactin-producing pituitary tumor (*n* = 10, ROR 51.64, PRR 51.63, IC 5.60, EBGM 48.62), lactation disorder (*n* = 21, ROR 44.86, PRR 44.85, IC 5.41, EBGM 42.56), retrograde ejaculation (*n* = 30, ROR 44.68, PRR 44.66, IC 5.41, EBGM 42.39), and auditory hallucination (*n* = 559, ROR 38.95, PRR 38.60, IC 5.21, EBGM 36.90). These signals may necessitate refinement in subsequent updates to the drug label.

**Table 3:** The top signal intensity of ADEs of paliperidone palmitate ranked by EBGM at the PTs level

System Organ Class	PTs	Case reports	ROR(95%CI)	PRR(χ2)	IC(IC025)	EBGM(EBGM05)
Investigations	Blood prolactin	3	222.49(62.07-797.55)	222.48(519.71)	7.45(5.83)	175.02(60.14)
Investigations	Blood prolactin increased	802	216.82(200.52-234.45)	213.94(134675.78)	7.41(7.29)	169.7(158.96)
Psychiatric disorders	Psychosexual disorder*	36	132.96(93.48-189.13)	132.88(4052.06)	6.84(6.33)	114.41(85.2)
Psychiatric disorders	Psychotic symptom*	220	123.62(107.26-142.49)	123.18(23163.82)	6.74(6.54)	107.15(95.14)
Investigations	Blood prolactin abnormal	82	93.42(74.33-117.42)	93.29(6718.79)	6.39(6.06)	83.82(69.23)
Psychiatric disorders	Schizoaffective disorder*	144	88.87(74.81-105.56)	88.66(11257.1)	6.32(6.07)	80.06(69.32)
Reproductive system and breast disorders	Galactorrhoea	531	84.07(76.86-91.95)	83.33(39195.68)	6.24(6.11)	75.7(70.23)
Psychiatric disorders	Schizophrenia	837	74.85(69.71-80.37)	73.82(55149.08)	6.08(5.98)	67.78(63.86)
Social circumstances	Refusal of treatment by patient*	319	66.33(59.16-74.36)	65.98(18888.7)	5.93(5.77)	61.12(55.54)
General disorders and administration site conditions	Injection site cyst	18	63.31(39.19-102.28)	63.29(1024.09)	5.88(5.19)	58.81(39.37)
Reproductive system and breast disorders	Breast discharge	94	61.19(49.62-75.48)	61.1(5169.94)	5.83(5.52)	56.91(47.75)
Psychiatric disorders	Blunted affect*	35	60.65(43.02-85.51)	60.62(1910.32)	5.82(5.32)	56.5(42.38)
Infections and infestations	Application site abscess	3	59.69(18.48-192.77)	59.69(161.32)	5.8(4.29)	55.69(20.88)
Social circumstances	Caffeine consumption*	3	56.92(17.66-183.47)	56.91(154.05)	5.74(4.23)	53.27(20)
Neoplasms benign, malignant and unspecified (incl cysts and polyps)	Prolactin-producing pituitary tumour*	10	51.64(27.25-97.85)	51.63(466.95)	5.6(4.71)	48.62(28.48)
Psychiatric disorders	Thought insertion*	3	50.99(15.88-163.7)	50.99(138.37)	5.59(4.09)	48.04(18.1)
Injury, poisoning and procedural complications	Exposure to contaminated device*	14	50.1(29.2-85.95)	50.09(634.59)	5.56(4.79)	47.25(30.08)
Social circumstances	Poor personal hygiene*	23	49.26(32.34-75.05)	49.25(1025.23)	5.54(4.93)	46.5(32.7)
General disorders and administration site conditions	Injection site nodule	483	45.59(41.59-49.99)	45.24(19800)	5.42(5.29)	42.91(39.73)
Reproductive system and breast disorders	Lactation disorder*	21	44.86(28.91-69.61)	44.85(853.32)	5.41(4.78)	42.56(29.47)
Reproductive system and breast disorders	Retrograde ejaculation*	30	44.68(30.94-64.53)	44.66(1213.98)	5.41(4.87)	42.39(31.17)
Psychiatric disorders	Manic symptom*	4	44.1(16.12-120.61)	44.1(159.83)	5.39(4.06)	41.89(18.05)
Reproductive system and breast disorders	Breast engorgement	10	43.17(22.85-81.55)	43.16(391.16)	5.36(4.47)	41.04(24.11)
Investigations	Spermatozoa abnormal*	10	41.42(21.94-78.18)	41.41(375.29)	5.3(4.41)	39.46(23.19)
Psychiatric disorders	Boredom*	14	41.09(24.02-70.3)	41.08(521.24)	5.29(4.53)	39.16(24.99)
Social circumstances	Imprisonment*	67	39.28(30.74-50.2)	39.24(2382.04)	5.23(4.87)	37.48(30.53)
Psychiatric disorders	Hallucination, auditory*	559	38.95(35.77-42.42)	38.6(19555.05)	5.21(5.08)	36.9(34.36)
Psychiatric disorders	Soliloquy*	30	37.79(26.2-54.49)	37.77(1026.3)	5.18(4.65)	36.14(26.6)
Psychiatric disorders	Attention-seeking behaviour*	5	35.16(14.36-86.08)	35.16(159.1)	5.08(3.87)	33.75(15.96)
General disorders and administration site conditions	Administration site induration	3	34.96(11.01-111.04)	34.96(94.9)	5.07(3.59)	33.57(12.76)
Investigations	Sperm concentration zero*	3	32.63(10.29-103.48)	32.63(88.45)	4.97(3.49)	31.41(11.96)
Psychiatric disorders	Flat affect*	57	32.16(24.68-41.91)	32.13(1654.32)	4.95(4.57)	30.95(24.8)
Nervous system disorders	Akathisia	424	31.99(29.02-35.26)	31.77(12163.93)	4.94(4.79)	30.61(28.22)
Psychiatric disorders	Psychotic disorder	827	31.59(29.46-33.88)	31.17(23274.97)	4.91(4.81)	30.06(28.36)
Psychiatric disorders	Neglect of personal appearance*	7	30.22(14.21-64.25)	30.21(190.66)	4.87(3.83)	29.17(15.52)
Psychiatric disorders	Emotional poverty*	53	29.95(22.76-39.39)	29.92(1429.19)	4.85(4.45)	28.9(22.97)
Psychiatric disorders	Automatism*	6	29.49(13.06-66.59)	29.49(159.36)	4.83(3.72)	28.49(14.41)
Nervous system disorders	Amimia*	3	29.14(9.21-92.17)	29.13(78.7)	4.82(3.34)	28.16(10.74)
Infections and infestations	Injection site abscess	64	28.91(22.53-37.1)	28.88(1663.49)	4.8(4.44)	27.92(22.66)
Metabolism and nutrition disorders	Hypermetabolism*	13	26.52(15.26-46.07)	26.51(309.11)	4.68(3.9)	25.71(16.19)
Endocrine disorders	Hyperprolactinaemia	178	25.97(22.37-30.16)	25.9(4129.97)	4.65(4.43)	25.13(22.18)
Nervous system disorders	Drooling	157	24.96(21.29-29.26)	24.9(3494.99)	4.6(4.36)	24.19(21.18)
Psychiatric disorders	Delusion*	348	24.7(22.2-27.49)	24.57(7639.01)	4.58(4.42)	23.88(21.83)
Reproductive system and breast disorders	Breast enlargement	82	24.41(19.59-30.41)	24.38(1785.1)	4.57(4.25)	23.7(19.72)
Metabolism and nutrition disorders	Water intoxication*	11	24.32(13.35-44.31)	24.32(238.83)	4.56(3.72)	23.64(14.31)
Reproductive system and breast disorders	Amenorrhoea	369	23.56(21.24-26.14)	23.42(7702.02)	4.51(4.36)	22.8(20.9)
Nervous system disorders	Patient elopement*	6	23.42(10.4-52.73)	23.42(125.18)	4.51(3.4)	22.79(11.56)
Psychiatric disorders	Paranoia*	349	22.54(20.26-25.08)	22.42(6952.09)	4.45(4.29)	21.84(19.98)
Investigations	Blood prolactin decreased*	4	21.78(9.49)	4.44(3.13)	22.35(79.4)	22.35(8.28-60.35)
Eye disorders	Oculogyric crisis	42	20.05(14.76-27.23)	20.04(741.44)	4.29(3.85)	19.58(15.15)

The asterisks indicate unexpected signals that are not listed in the drug label. *ADEs* Adverse drug events, *EBGM* Empirical Bayesian Geometric Mean, *PTs* Preferred terms

Paliperidone palmitate is frequently used in combination with other medications, including depakote, risperidone, seroquel, and olanzapine. After excluding reports involving the concomitant use of additional drugs, we identified a total of 9,953 reports, corresponding to 16,346 adverse events. Persistent potential adverse reactions associated with paliperidone palmitate included weight gain, elevated prolactin levels, psychotic disorders, galactorrhea, amenorrhea, akathisia, extrapyramidal disorders, suicidal ideation, hyperprolactinemia, and erectile dysfunction (Table S3).

### Gender-based difference in risk signals for paliperidone palmitate

**Fig. 2:**
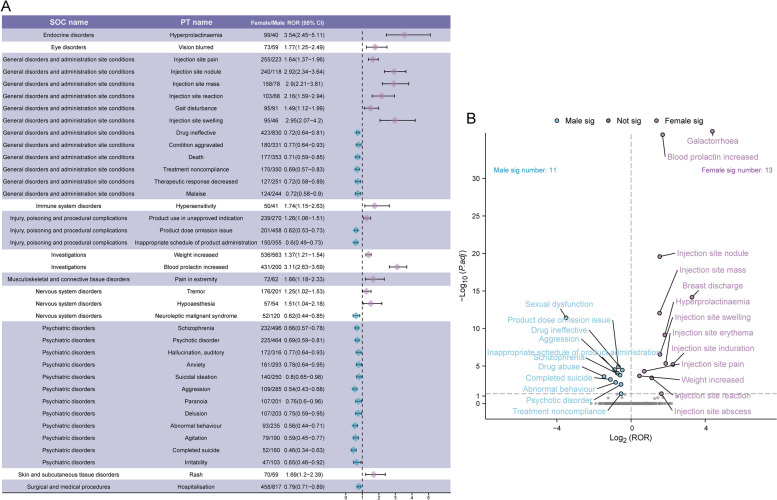
Gender-differentiated risk signal analysis of paliperidone palmitate-associated adverse drug events (ADEs). **A** Reporting odds ratios (ROR) with 95% confidence intervals (CI) for identified gender-related ADEs with reported cases no less than 40. **B** Gender-differentiated risk signal volcano plot for paliperidone palmitate. The x-axis represents the log2-transformed ROR values, while the y-axis depicts the -log10-transformed adjusted *P*-values. Significant signals are emphasized with prominent colors and annotated accordingly. The *P*-value is adjusted with Bonferroni correction method. Two of the PTs displayed values above the upper limit of the figure, including blood prolactin increased, and galactorrhea

There may be gender differences in the types of ADEs, influenced by factors such as physiological structure, hormone levels, and pharmacokinetics [22]. To investigate potential gender differences in the signals of adverse reactions to paliperidone palmitate, we employed the ROR method to identify 89 PTs that occurred disproportionately in males and females, which were then categorized by SOC. All data results are provided in Table S4. We present a forest plot of the 38 signals with a case count of at least 40 (Fig. 2A). Compared to females, males were more likely to experience psychiatric disorders, including schizophrenia, psychotic disorders, anxiety, suicidal ideation, and paranoia. Additionally, high-risk signals for males included neuroleptic malignant syndrome, malaise, and hospitalization. In contrast, high-risk signals for females included hyperprolactinemia, weight gain, blurred vision, hypersensitivity, tremor, rash, and injection site reactions.

To further visualize the gender-specific results of paliperidone palmitate-related ADE signals, we created a volcano plot (Fig. 2B). Each point in the plot represents an ADE associated with paliperidone palmitate, with statistically significant signals labeled. Eleven significant signals were identified in males, including drug ineffective, schizophrenia, aggression, sexual dysfunction, drug abuse, psychotic disorder, and completed suicide. Thirteen significant signals were identified in women, including weight increased, galactorrhea, elevated blood prolactin, hyperprolactinemia, injection site erythema, and others.

Moreover, recognizing the potential confounding impact of baseline information on the outcomes of disproportionality analyses, sensitivity analyses incorporating gender were conducted to enhance result reliability [78]. Figures S2A and S2B depict the top 25 PTs ranked by the number of reports for males and females, respectively. Specifically, signals included erectile dysfunction, abnormal behavior, paranoia, delusion, gynecomastia, and depression have higher reporting ranks in males. Conversely, galactorrhea, amenorrhea, injection site nodules, injection site mass, extrapyramidal disorders, and hallucinations have higher reporting ranks in females. Additionally, Figure S2C illustrates 16 signals common to both genders.

### TTO analysis of paliperidone palmitate-related ADEs

**Fig. 3:**
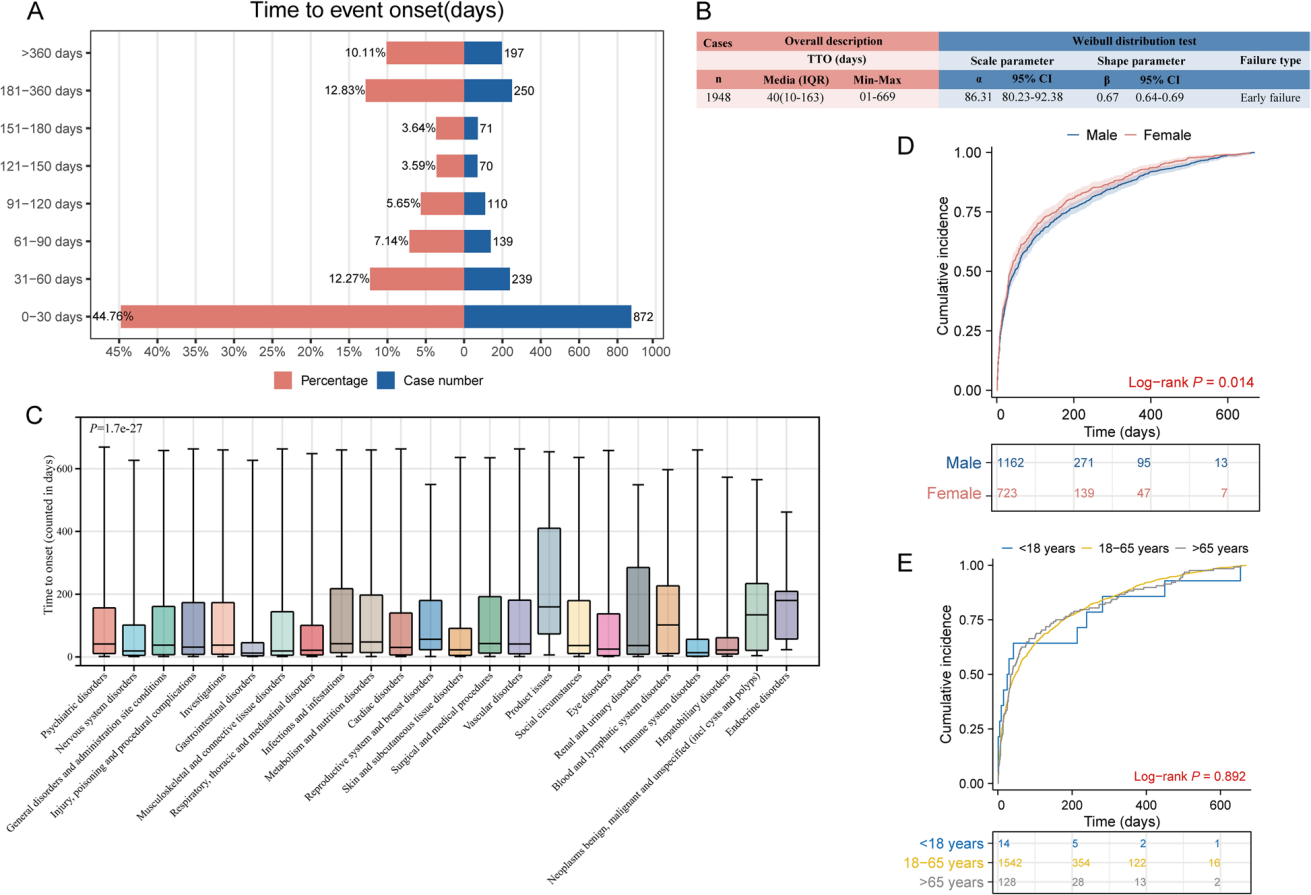
Time to onset (TTO) analysis (counted in days) of paliperidone palmitate-related ADEs. **A** The bar charts illustrate the quantity and proportion of TTO reports within varying time intervals. **B** Overall description and Weibull distribution test for all TTO reports. **C** Box plot displays the TTO of ADEs at the SOC level. Black bar within the stick: median TTO; lower end of the stick: 1/4 quantile of the TTO; upper end of the stick: 3/4 quantile of the TTO. **D** Comparison between cumulative incidences of ADEs between males and females E. Comparison of cumulative incidences of ADEs across different age groups. IQR, interquartile range; Min, minimum; Max: maximum

Understanding the TTO of ADEs is crucial for physicians to make informed decisions. After excluding inaccurate, missing, or erroneous reports, a total of 1,948 (7.0%) TTO reports were gathered. As depicted in Fig. 3A, a majority of cases occurred within the initial month (*n* = 872, 44.76%). Despite this, ADEs remained probable even after one year of paliperidone palmitate treatment (*n* = 197, 10.11%). The median time to onset for all ADEs related to paliperidone palmitate was 40 days (interquartile range [IQR] 10–163 days) (Fig. 3B). The comprehensive analysis revealed a shape parameter (β) of 0.67 with an upper limit of its 95% confidence interval (CI) at 0.69. These values being < 1 suggest a decrease in ADE prevalence over time. In a detailed assessment of ADE timing, the TTO at the SOC level was analyzed (Fig. 3C and Table S5). Gastrointestinal disorders linked to paliperidone palmitate exhibited the shortest median time to onset at 12 days, while endocrine disorders had the longest median time to onset at 180 days.

Age-related changes in pharmacokinetics and pharmacodynamics render elderly patients particularly susceptible to ADEs [16]. Furthermore, the timing of ADE may be influenced by gender, reflecting the effects of sex hormones, metabolic differences, and behavioral factors [12, 68]. To address these considerations, we stratified the TTO analyses by both gender and age groups. The Kaplan-Meier curves presented in Fig. 3D and E illustrated the cumulative incidence of ADEs of paliperidone palmitate within gender subgroups and age subgroups. We observed that ADEs occurred significantly earlier in females than in males (*P* = 0.014). However, this difference was not present in the subgroup analysis based on age (*P* = 0.892).

### TTO analysis at the PT level and long-term safety assessment

**Fig. 4:**
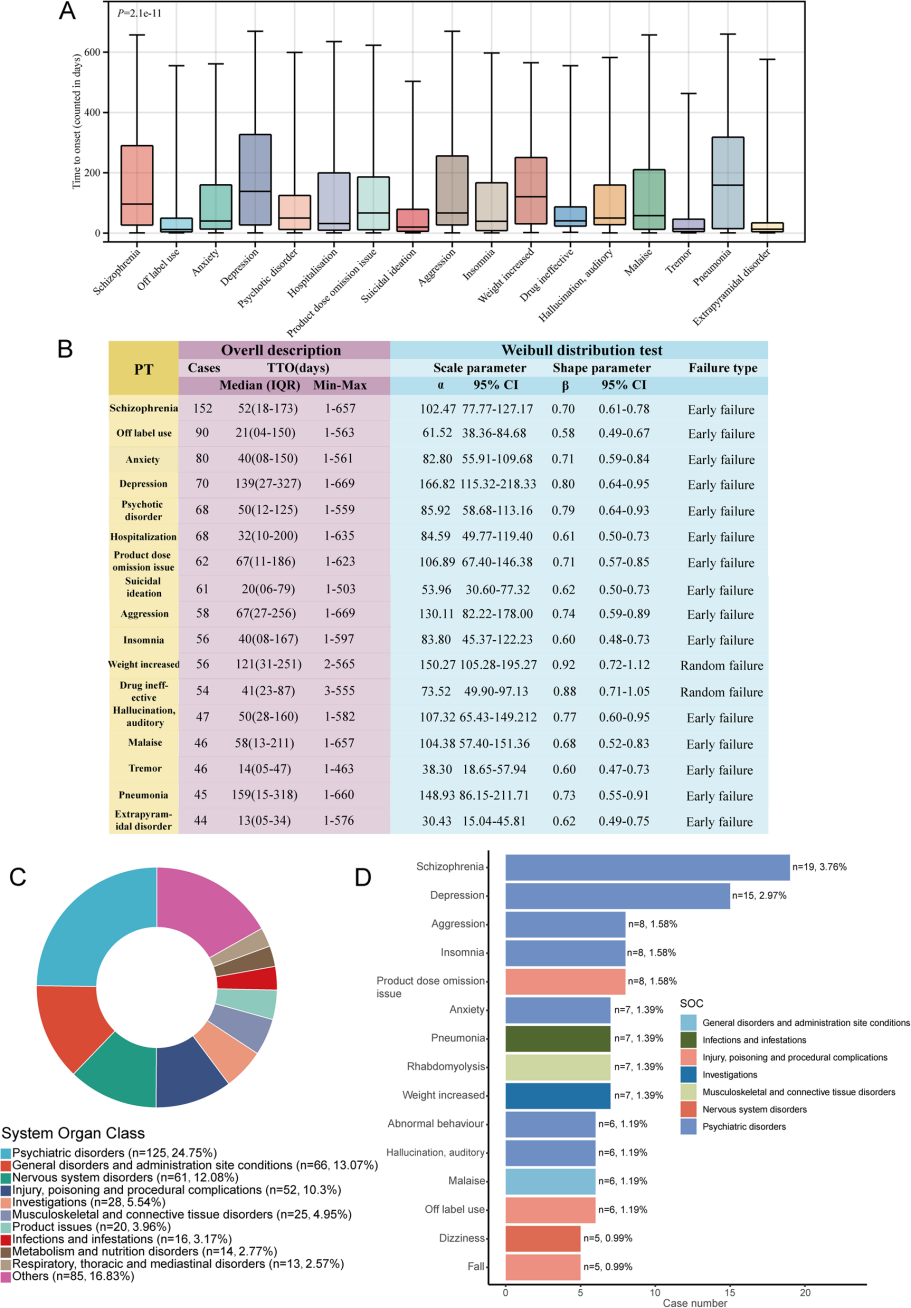
Time-to-onset (TTO) analyses at the preferred term (PT) level and for adverse drug event (ADE) reports occurring after 360 days. **A** Box plot displays the TTO of ADEs (no less than 40 cases) at the PT level. Black bar within the stick: median TTO; lower end of the stick: 1/4 quantile of the TTO; upper end of the stick: 3/4 quantile of the TTO. **B** Overall description and Weibull distribution test for TTO reports at the PT level. **C** Donut plot illustrating the distribution of System Organ Classes (SOC) for TTO reports after 360 days. **D** Distribution of preferred terms (PTs) associated with each ADE in the safety report after 360 days. The bar chart lists the top 15 PTs for the number of cases

Given the considerable variability in the TTO of ADEs, we conducted an analysis at the PT level to provide more precise insights. Data from over 40 reports covering 17 PTs were collected and aggregated. A significant difference in TTO across PTs was observed (*P* = 2.1e-11) (Fig. 4A). Among the PTs analyzed, those with the shortest median onset times (MOTs) included extrapyramidal disorder (MOT: 13 days), tremor (MOT: 14 days), and suicidal ideation (MOT: 20 days). Conversely, PTs with the longest MOTs included pneumonia (MOT: 159 days), depression (MOT: 139 days), and weight increased (MOT: 121 days) (Fig. 4B). Furthermore, the Weibull distribution analysis revealed that two PTs exhibited a random failure type, indicating consistent occurrence over time, whereas the remaining 15 PTs showed an early failure type. To assess long-term safety, we analyzed all adverse events occurring after 360 days of paliperidone palmitate administration. Among 505 TTO reports with onset times exceeding 360 days, the most frequently reported SOC was “general disease and medication site conditions” (*n* = 115, 22.8%), followed by “psychiatric disorders” (*n* = 125, 24.75%) and “general disorders and administration site conditions” (*n* = 66, 13.07%) (Fig. 4C). The most commonly reported PTs beyond 360 days included schizophrenia, depression, aggression, insomnia, and product dose omission issues (Fig. 4D).

### External validation in JADER database

**Fig. 5:**
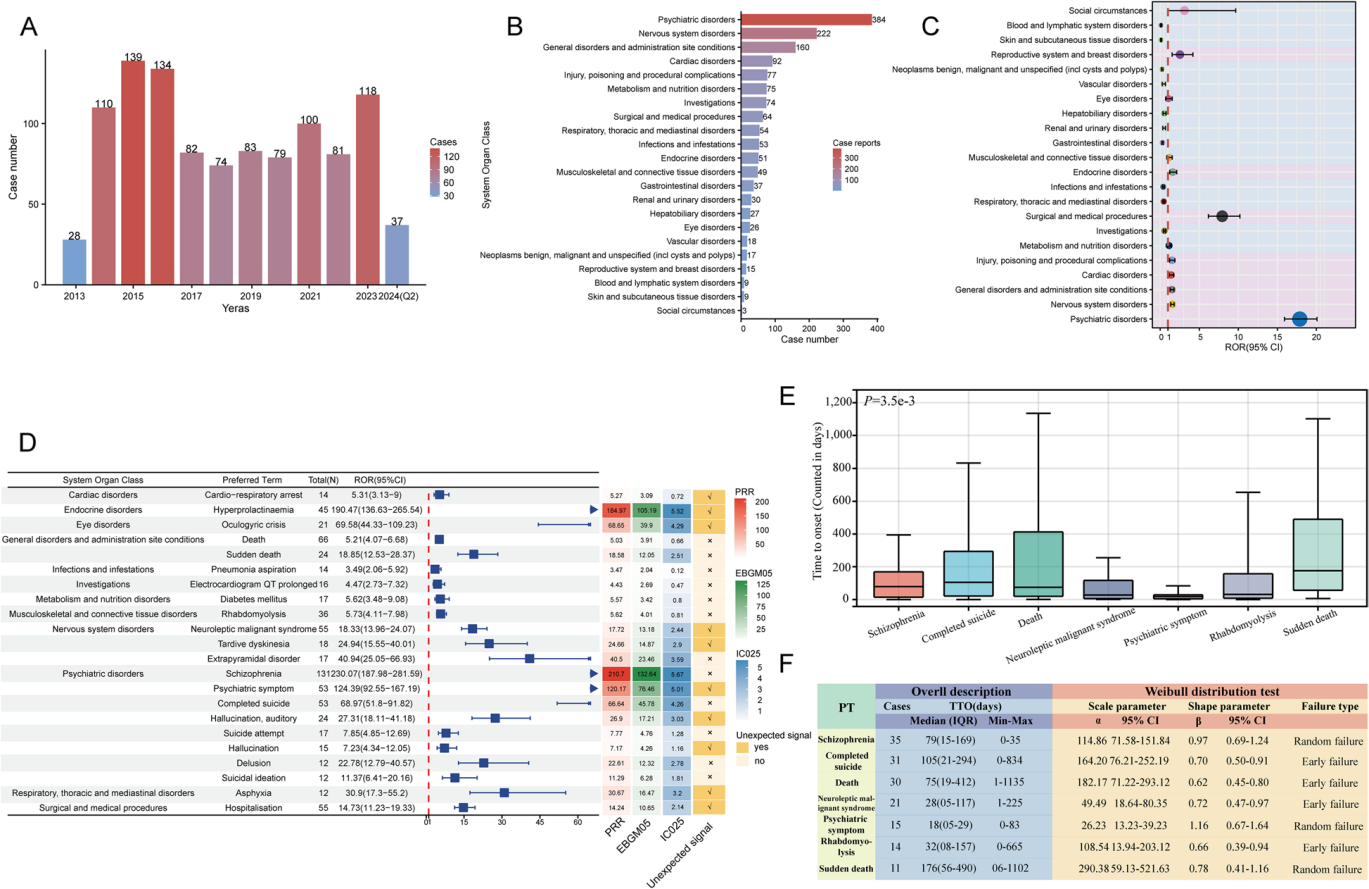
External validation from the JADER database. **A**. Temporal distribution of adverse drug event (ADE) reports spanning from 2013 to the second quarter of 2024 **B**. Bar chart illustrating the cases of ADE reports stratified by System Organ Class (SOC) level. **C**. Signal detection at the SOC level: A forest plot representing Reporting Odds Ratio (ROR) values along with their 95% confidence intervals (CIs). A purple background denotes signals exceeding the positive threshold, while a blue background indicates those that do not. **D**. Signal detection at the preferred term (PT) level: The forest plot and accompanying heatmap depict signal values for PTs with at least 10 reported cases. E. Time to onset (TTO) analysis at the PT level: Box plots illustrate the distributional characteristics of TTO for various PTs. F. Overall analysis Weibull distribution test of TTO reports. JADER, Japanese Adverse Drug Event Report

We validated the FAERS results using data from the JADER database. Between January 2013 and June 2024, a total of 1,065 reports of paliperidone palmitate-related ADEs were collected (Fig. 5A). The highest number of reports (*n* = 139) occurred in 2015 (Fig. 5A). The distribution of ADEs across SOCs is shown in Fig. 5B, with the top three SOCs being psychiatric disorders (*n* = 384), nervous system disorders (*n* = 222), and general disorders and administration site conditions (*n* = 160).

Signal detection at the SOC level revealed that eight SOCs exceeded the positive threshold of the ROR algorithm. These SOCs included psychiatric disorders, nervous system disorders, general disorders and administration site conditions, cardiac disorders, injury, poisoning, and procedural complications, surgical and medical procedures, endocrine disorders, and reproductive system and breast disorders (Fig. 5C, Table S6). At the PT level, 51 positive signals were identified, of which 22 PTs were reported in 10 or more cases. These PTs were further categorized by SOC (Fig. 5D). PTs with a high number of cases and strong signal values included hyperprolactinemia (*n* = 45, ROR 190.47, PRR 184.97, EBGM05 105.19, IC025 5.52), oculogyric crisis (*n* = 21, ROR 69.58, PRR 68.65, EBGM05 39.90, IC025 4.29), neuroleptic malignant syndrome (*n* = 55, ROR 18.33, PRR 17.73, EBGM05 13.18, IC025 2.44), schizophrenia (*n* = 131, ROR 230.07, PRR 210.70, EBGM05 134.64, IC025 5.67), psychiatric symptoms (*n* = 45, ROR 124.39, PRR 120.17, EBGM05 76.46, IC025 5.01), and completed suicide (*n* = 53, ROR 68.97, PRR 66.64, EBGM05 45.78, IC025 4.26).

A comparison of JADER and FAERS identified 35 overlapping positive signals, including schizophrenia, akathisia, extrapyramidal disorder, erectile dysfunction, neuroleptic malignant syndrome, hyperprolactinemia, and rhabdomyolysis (Table S6). JADER-specific signals included diabetes mellitus, electrocardiogram QT prolongation, increased blood glucose, and acute myocardial infarction.

TTO analysis at the PT level revealed that psychiatric symptoms had the shortest MOT of 18 days, whereas sudden death had the longest MOT of 176 days. A significant difference in TTO was observed among the seven PTs analyzed (*P* = 3.5e-3) (Fig. 5E). The Weibull distribution analysis indicated that schizophrenia and psychiatric symptoms followed a random failure type, whereas all other PTs exhibited an early failure type (Fig. 5F).

## Discussion

Given the diverse patient population and complexities in real-world scenarios, data derived from clinical trials may not fully reflect real-world experiences. Consequently, this study seeks to uncover potential new ADEs and gender disparities associated with paliperidone palmitate, and to scrutinize the TTO using an extensive analysis of extensive data from the FAERS database. These results have the potential to inform updates to drug labeling and offer fresh insights for the judicious utilization of paliperidone palmitate in clinical settings.

The results of our study revealed higher reported frequencies of ADEs associated with paliperidone palmitate in males (45.3%) compared to females (31.0%). This discrepancy may be attributed to the elevated prevalence of schizophrenia, the drug’s primary indication, among males as opposed to females [32]. Regrettably, age and weight data are largely unknown. The principal reporters were health professionals and consumers, underscoring the varied roles of stakeholders in ADE surveillance. Analysis of the annual distribution of paliperidone palmitate-related ADE reports indicated a peak of 3,010 cases in 2017, with over 1,800 reported cases annually since then. These findings highlight the widespread usage and efficacy of paliperidone palmitate in clinical settings, emphasizing the importance of monitoring its adverse effects.

### Common and newly identified ADEs

As a psychotropic medication, the ADEs associated with paliperidone palmitate predominantly involve injury, poisoning and procedural complications, general disorders and administration site conditions, and psychiatric disorders, aligning with its pharmacologic effects and prescribed uses. The results of the disproportionality analysis revealed ten significant signals at the SOC level (Table S1), some of which are explicitly mentioned in the drug label, validating the reliability of our findings. Notably, heightened signal intensities were observed for “psychiatric disorders”, “social circumstances”, “surgical and medical procedures”, and “reproductive system and breast disorders,” indicating a strong association between ADEs in these categories and the clinical administration of the drug. Clinicians should exercise heightened vigilance during treatment and acknowledge the potential risks associated with paliperidone palmitate.

The three PTs with the highest reported frequencies in FAERS were “off-label use,” “drug ineffective,” and “hospitalization,” as highlighted in FigureS1. Particularly noteworthy, “off label use” (*n* = 2,616) emerged as the most prevalent among all positive PTs, followed by “hospitalization” (*n* = 1,662) and “weight increased” (*n* = 1,286). These findings underscore the significance of standardizing medication usage and the necessity for consistent monitoring of patient responses to therapy. Among the PTs consistent with the drug label, “weight increased” (*n* = 1,286), “schizophrenia” (*n* = 837), and “psychotic disorder” (*n* = 827), stood out as the three most common. In a noninferiority study involving different doses of paliperidone palmitate, 21% of patients experienced weight gain [61]. While psychotic disorder and schizophrenia are typically deemed rare serious ADEs in clinical trials [5, 14], our data indicated a higher reporting rank. It is essential to consider that these ADEs may not exclusively be drug-induced but could stem from inadequate therapeutic control or pre-existing conditions, especially considering some treatment-resistant schizophrenia cases [63]. Our results raise concerns regarding medication safety, suggesting the potential necessity for additional assessment tools or questionnaires to mitigate severe ADEs.

Through a real-world analysis of paliperidone palmitate, “blood prolactin increased” showed a higher signal value (*n* = 802, ROR 216.82, PRR 213.94, EBGM05 158.96, IC025 7.29). Given that paliperidone is the primary active metabolite of risperidone, the mechanism through which paliperidone induces elevated prolactin levels may mirror that of risperidone, involving D2 dopamine receptor antagonism [56]. Elevated prolactin can lead to decreased gonadal steroid levels and hypogonadotropic hypogonadism by suppressing pituitary hormone and follicle-stimulating hormone release, potentially resulting in various ADEs in both genders [71]. ADEs stemming from hyperprolactinemia are dynamic processes rather than isolated incidents.

The seven most prevalent unexpected ADEs in psychiatric disorders, based on report numbers, were auditory hallucinations (*n* = 559, ROR 38.95, PRR 38.60, EBGM50 34.36, IC025 5.08), depression (*n* = 504, ROR 2.25, PRR 2.24, EBGM05 2.08, IC025 1.03), aggression (*n* = 434, ROR 9.45, PRR 9.38, EBGM05 8.58, IC025 3.08), suicidal ideation (*n* = 419, ROR 4.86, PRR 4.84, EBGM05 4.44, IC 2.13), hallucination (*n* = 351, ROR 4.98, PRR 4.96, EBGM05 4.52, IC 2.15), paranoia (*n* = 349, ROR 22.54, PRR 22.42, EBGM05 19.98, IC025 4.29), and delusion (*n* = 348, ROR 24.70, PRR 24.57, EBGM05 21.83, IC025 4.42). Hallucinations auditory, aggression, paranoia, and delusions are typically seen as exacerbations or relapses of underlying disorders, potentially linked to inappropriate use of antipsychotic medications [58]. The notable frequency of suicidal ideation is a significant concern, especially given the high mortality rate among schizophrenia patients [39]. Unexpected suicide-related PTs identified include completed suicide (*n* = 231, ROR 2.90, PRR 2.90, EBGM05 2.59, IC025 1.34) and suicide attempt (*n* = 166, ROR 2.93, PRR 2.93, EBGM05 2.57, IC025 1.32). While meta-analyses of randomized controlled trials have indicated that LAIs do not substantially raise the risk of suicide death [35], establishing a definitive association between LAIs and suicidal ideation has been challenging due to study duration limitations and population constraints. An initial marketing alert for paliperidone palmitate reported 32 deaths, including 7 suicides [24]. The identification of numerous ADEs in psychiatric disorders signals a heightened risk of suicidal ideation, necessitating thorough mental health evaluations by physicians and continual monitoring for patients under prolonged usage of this medication. Sudden death (*n* = 66, ROR 7.11, PRR 7.10, EBGM05 5.76, IC025 2.46) represents a serious and unexpected ADE that necessitates close attention. This outcome may be linked to the patient’s underlying comorbidities, such as cardiovascular disease. Paliperidone palmitate has been associated with arrhythmias, including abnormalities in the electrocardiogram QT interval and repolarization. For patients with such pre-existing conditions, it is crucial to conduct regular electrocardiogram monitoring to detect potential risks.

The analysis also unveiled unexpected signals with high signal values, including “psychosexual disorder” (*n* = 36, ROR 132.96, PRR 132.88, EBGM05 85.2, IC025 6.33), “lactation disorder” (*n* = 21, ROR 44.86, PRR 44.85, EBGM05 29.47, IC025 4.78), and “retrograde ejaculation” (*n* = 30, ROR 44.86, PRR 44.85, EBGM05 29.47, IC025 4.78). These ADEs should not be underestimated, as they can impact patients’ quality of life, potentially affecting adherence. Consequently, distinguishing between gender-specific preferred terms is warranted. The clinical manifestations of hyperprolactinemia and hypogonadism may pose diagnostic challenges [65]; therefore, in addition to monitoring prolactin level changes, recognizing associated ADE symptoms is crucial. Notably, our study outcomes present a comprehensive list detailing the case numbers and signal values of paliperidone palmitate-related ADEs related to reproductive system and breast disorders. This valuable insight aids in early recognition of these intricate ADEs, contributing to the safer use of the medication.

One of the potential long-term consequences of antipsychotic-induced hyperprolactinemia is the development of pituitary tumors. We also identified prolactin-producing pituitary tumors (*n* = 10, ROR 51.64, PRR 51.63, EBGM05 28.48, IC025 4.71) with positive signal values, albeit in relatively small numbers. Strikingly, there have been no prior reports of pituitary tumors linked to paliperidone palmitate use. Nevertheless, a female adolescent patient was diagnosed with a pituitary adenoma during psychiatric treatment with risperidone [57]. It is crucial to acknowledge the shared pharmacologic mechanisms of these medications. This potential adverse drug event demands attention, particularly considering the limitations in clinical study follow-up duration. Our findings indicate a disproportionate reporting of pituitary tumor cases associated with paliperidone palmitate, warranting further investigation in prospective studies.

### Adherence-related ADEs

The long-term therapeutic efficacy of treatment is highly dependent on medical adherence, as poor adherence significantly increases the risk of disease recurrence. Compared to oral antipsychotics, paliperidone palmitate has been associated with lower rates of rehospitalization and emergency department visits [11, 55]. Previous studies have demonstrated that LAIs offer better compliance than oral antipsychotics [40], with particular emphasis on the impact of switching between different formulations of paliperidone palmitate [41], suggesting that the route and frequency of administration are important factors. However, the EULAST trial, which included 533 patients with early-stage schizophrenia, found no significant difference in the duration of all-cause discontinuation between the combined oral and LAI treatment groups [75]. The level of compliance specifically with paliperidone palmitate has not been adequately addressed. Our study highlights several critical issues related to adherence, including product dose omission (*n* = 1,044, ROR 4.41, PRR 4.35, EBGM05 4.12, IC025 2.02), incorrect dose administration (*n* = 798, ROR 3.83, PRR 3.80, EBGM 3.57, IC025 1.82), and inappropriate administration schedules (*n* = 661, ROR 4.04, PRR 4.01, EBGM05 3.75, IC025 1.89). These findings underscore non-compliance might serve as a significant barrier to the effective use of paliperidone palmitate in real-world settings. Improving patient adherence is crucial to enhancing treatment outcome [8, 9]. Key strategies include the development of individualized treatment plans that are tailored to each patient’s needs and preferences, alongside patient education that facilitates clear communication and understanding of the benefits and risks of treatment. Additionally, incorporating modern technologies such as digital reminders and remote monitoring through telehealth platforms can further support adherence. Building trust between patients and healthcare providers, along with active involvement of family members and community programs, can further reinforce adherence through continuous support and encouragement [43].

### Gender-specific differences in drug safety

Erectile dysfunction in males and amenorrhea in females, both positive adverse effects, are consistent with the established side effect profile of paliperidone palmitate. Previous research highlighted erectile dysfunction as the most common sexual dysfunction in males with schizophrenia, while females commonly experienced orgasmic dysfunction and amenorrhea [37]. Addressing sexual dysfunction can enhance medication adherence by encouraging open communication between patients and healthcare providers. Healthcare professionals should be vigilant for ADEs like increased blood prolactin levels causing gynecomastia in males and galactorrhea in females when administering paliperidone palmitate. This may be related to the different hormone levels and the role of the sex organs in males and females. A meta-analysis involving individual participant data and clinical study reports found three gynecomastia events occurred, two of which were associated with paliperidone palmitate [27]. Gender differences ADE signal analysis is essential when evaluating drug safety, given the observed different gender rations of baseline information. As shown in Fig. 2A, females are more likely to experience ADEs related to injection sites and elevated prolactin levels. To further explore the relationship between gender and ADEs, we validated these findings by adjusting the *P*-value and found that female patients are predominantly associated with ADEs linked to prolactin and weight increase. Previous studies have reported that females taking antipsychotic medications typically have higher prolactin levels than males, making them more susceptible to prolactin-related ADEs [7, 25]. Women may be more attuned to concerns about their appearance, and as a result of heightened societal expectations, they are likely to experience increased intrinsic stress [40]. This may make them more prone to reporting ADEs related to body image and injection sites. In contrast, males may place greater importance on sexual function than females, given the distinct expectations they have regarding sexual performance. It is important to note that men also experience ADEs associated with more severe outcomes, such as death and completed suicide. These findings highlight the need for attention to gender-specific ADEs in clinical practice. However, further clinical evidence is required to validate these observations.

### Time to onset analysis

The second-generation LAI antipsychotic, paliperidone palmitate, utilized to mitigate nonadherence and relapse rates in patients with schizophrenia spectrum disorders, faces limited utilization due to its ADEs despite its promising efficacy [48]. Understanding the temporal relationship between medications and ADEs is critical for assessing drug safety, aiding in the prevention or early detection of ADEs. Our study revealed that ADEs linked with paliperidone palmitate primarily occurred within the initial month, facilitating early ADE monitoring for long-term medication regimes. Nevertheless, certain ADEs may manifest up to a year later, underscoring the necessity to remain vigilant for cumulative drug metabolism effects. Monitoring the drug’s plasma concentrations could offer an alternative approach to enhance paliperidone palmitate tolerability, albeit with substantial individual variability in plasma levels [62]. A recent case report on paliperidone palmitate overdose demonstrated that plasma paliperidone concentrations remained at the lower end of the therapeutic range 2.5 years after the last dose [52]. Encouragingly, the Weibull distribution analysis indicated a decreasing likelihood of ADEs over time for paliperidone palmitate, a reassuring outcome for a medication requiring prolonged administration. While limited research has investigated the timing of paliperidone palmitate’s adverse effects across various organ systems, our study provides a comprehensive account of onset times at the SOC level. Notably, nervous system disorders associated with paliperidone palmitate exhibited a median onset time of 19 days. In a randomized, double-blind, non-inferiority study on extrapyramidal symptom (EPS)-related treatment-emergent adverse events (TEAEs), TTO was compared between paliperidone palmitate 3-monthly (PP3M) and paliperidone palmitate monthly (PP1M), with results indicating a median TTO of 17 days for all EPS-associated TEAEs during the open-label phase (PP1M)(Mathews et al., [45]. Although specific PTs onset times were not analyzed, our neurological ADEs onset time closely aligned with existing literature. Our findings highlight the necessity of establishing distinct follow-up and review timelines for ADEs within different SOCs, understanding that individual variabilities in drug response necessitate tailored monitoring strategies. We further investigated potential gender and age differences in the timing of ADEs associated with paliperidone palmitate and conducted subgroup analyses. The results demonstrated that ADEs occurred significantly earlier in females compared to males. However, no significant difference was observed in age subgroup analyses. Similarly, Chen et al. demonstrated that the MOT of immune-related adverse events was significantly longer in male patients compared to female patients following immune checkpoint inhibitor therapy [12]. This disparity may be attributed to gender-related differences in drug metabolism rates, physiological characteristics, and pharmacokinetic properties, including absorption, distribution, metabolism, and excretion [80]. These findings underscore the importance of considering gender differences in the timing of ADE onset during clinical practice to optimize patient care and safety.

### External validation and database comparisons

The baseline demographic characteristics reported by the JADER system were similar to those from the FAERS. Among all ADE reports collected by JADER, 54.7% were submitted by male reporters, compared to 42.8% by female reporters. This distribution aligns with the epidemiological profile of schizophrenia, where the male-to-female prevalence ratio is approximately 1.4, and males often experience a more severe disease course [49]. In terms of reported adverse events, JADER demonstrated a higher reporting rank of nervous system and cardiac disorders compared to FAERS. Notably, seven of the eight positive SOCs identified in JADER were also positive in FAERS, with cardiac disorders representing a JADER-specific positive SOC. Additionally, 35 positive signals identified in JADER overlapped with those in FAERS, including hyperprolactinemia, erectile dysfunction, and rhabdomyolysis. Hyperprolactinemia, a significant signal in JADER, is associated with various adverse hormonal effects, such as sexual dysfunction, gynecomastia, amenorrhea, and galactorrhea [15]. A clinical study from Japan demonstrated a positive correlation between serum prolactin levels and the daily dose of paliperidone in male schizophrenia patients [70]. Interestingly, while hyperprolactinemia is listed as a known adverse signal in FAERS, it is considered an unexpected signal in JADER, warranting attention for future updates to drug safety specifications. Antipsychotic-induced sexual dysfunction, a commonly underestimated adverse effect, significantly impacts quality of life and frequently contributes to treatment nonadherence [66]. A case report highlighted a young male patient in his 20s with schizophrenia who experienced erectile dysfunction and retrograde ejaculation approximately three months after initiating paliperidone palmitate therapy. These symptoms resolved following a switch to alternative treatment [44]. Proposed mechanisms for such adverse events include dysregulation of dopamine and serotonin (5-hydroxytryptamine) receptors, as well as neuroendocrine dysfunction [50]. Although antipsychotics are not commonly associated with rhabdomyolysis, several case reports have established a direct link between paliperidone palmitate use and the condition [2, 23, 36]. Rhabdomyolysis is a clinical syndrome characterized by skeletal muscle breakdown and the release of intracellular components into the bloodstream [28]. In cases of paliperidone palmitate-induced rhabdomyolysis, potential contributing factors include antagonism of serotonin receptors, impaired glucose uptake in muscle tissue, and myocardial changes. This condition is often linked to antipsychotic malignant syndrome and acute dystonic reactions [23]. We recommend close monitoring of creatine phosphokinase levels in patients at high risk for rhabdomyolysis (e.g., those presenting with acute dystonia) during paliperidone palmitate therapy. Early detection and intervention are crucial to prevent complications such as renal injury and life-threatening arrhythmias.

In addition, several signals specific to the JADER database were identified, including diabetes mellitus, electrocardiogram QT prolongation, elevated blood glucose levels, and acute myocardial infarction. Preclinical studies have demonstrated that paliperidone prolongs the QT interval by inhibiting human ether-à-go-go-related gene (hERG) potassium currents at clinically relevant concentrations [72]. Supporting this, a clinical study conducted among Japanese patients revealed a significant positive correlation between plasma paliperidone levels and corrected QT interval (QTc)(Suzuki et al., [67]. Further evidence of glucose-related adverse effects associated with paliperidone was observed in a 2-year, open-label, multicenter study in adolescents with schizophrenia. The study reported that 4.3% of patients (*n* = 14) experienced a change in fasting glucose levels from normal to high, while glucose-related adverse events occurred in 6 patients (1.5%) (Savitz et al., [60]. Additionally, short- and long-term clinical trials indicated that paliperidone significantly increased glucose concentrations compared to placebo [19]. Animal studies have provided mechanistic insights, showing that intravenous administration of paliperidone in rats induces hyperglycemia, possibly through hypothalamic AMP-activated protein kinase (AMPK) activation, which stimulates adrenaline secretion [76]. While the findings from JADER align with those of the FAERS, it is crucial to interpret these results in the context of potential differences in ethnicity, social background, and medical conditions between the two populations. Although the consistency between JADER and FAERS enhances the validity of the observed signals, such factors should be carefully considered when comparing and contrasting these pharmacovigilance databases.

### Limitations

This study utilized data from the FAERS and JADER databases to investigate ADE signals associated with paliperidone palmitate. By addressing the limitations of small sample sizes and short observation periods inherent in clinical trials, this approach provides a more comprehensive analysis. Additionally, integrating data from two independent sources enables cross-validation of ADEs and minimizes potential biases associated with reliance on a single database. Nonetheless, several limitations of this study should be acknowledged.

1. Differences in case reporting: A considerable disparity exists between the number of cases reported in the two databases, with JADER accounting for fewer than one-tenth of the cases reported in FAERS. Furthermore, while JADER is restricted to case reports, FAERS includes periodic reports, covering non-serious cases over extended periods [79]. These differences may have influenced the results of our analyses.
2. Limitations of spontaneous reporting systems: Both databases are subject to inherent limitations, including underreporting, incomplete reporting, and selective reporting [51]. Despite conducting sensitivity analyses, the lack of detailed patient clinical data—such as comorbidities, severity of underlying diseases, and concomitant medications—complicated our ability to control for confounding variables [46]. This limitation may have impacted the accuracy of our findings.
3. Potential influence of prior treatments: While we focused on paliperidone palmitate as the PS in ADE reports, adverse events during treatment may be influenced by prior exposure to oral antipsychotic agents. Cumulative effects from such prior treatments pose challenges in attributing ADEs solely to the injectable preparation [3].
4. Limitations of disproportionality analyses: Disproportionality analyses, while effective for assessing signal strength and establishing statistical relationships, are inherently limited. They do not quantify risk or establish causality, which constrains the interpretability of our findings [3].
5. Dose and route of administration: Due to limited drug-specific information, our analysis did not differentiate between varying doses of injectable paliperidone palmitate. Instead, it focused on the general adverse effects associated with the preparation. Moreover, the majority of reported cases (over 97%) involved intramuscular administration, leaving little room for analysis of alternative routes.
6. Population generalizability: The study primarily relied on data from the United States (FAERS) and Japan (JADER), potentially limiting the generalizability of the findings to populations with different demographic characteristics, healthcare practices, and prescribing patterns [42].
7. TTO reporting: The low rate of TTO reporting in FAERS (7.0%) significantly constrained our ability to conduct comprehensive analyses related to ADE timing. This necessitates caution when interpreting TTO findings.

In summary, while our study leverages robust datasets and rigorous methodologies, these limitations underscore the need for caution in interpreting the results. Future research, including detailed clinical studies and assessments, is warranted to validate the associations observed and further elucidate the safety profile of paliperidone palmitate.

## Conclusion

**Fig. 6:**
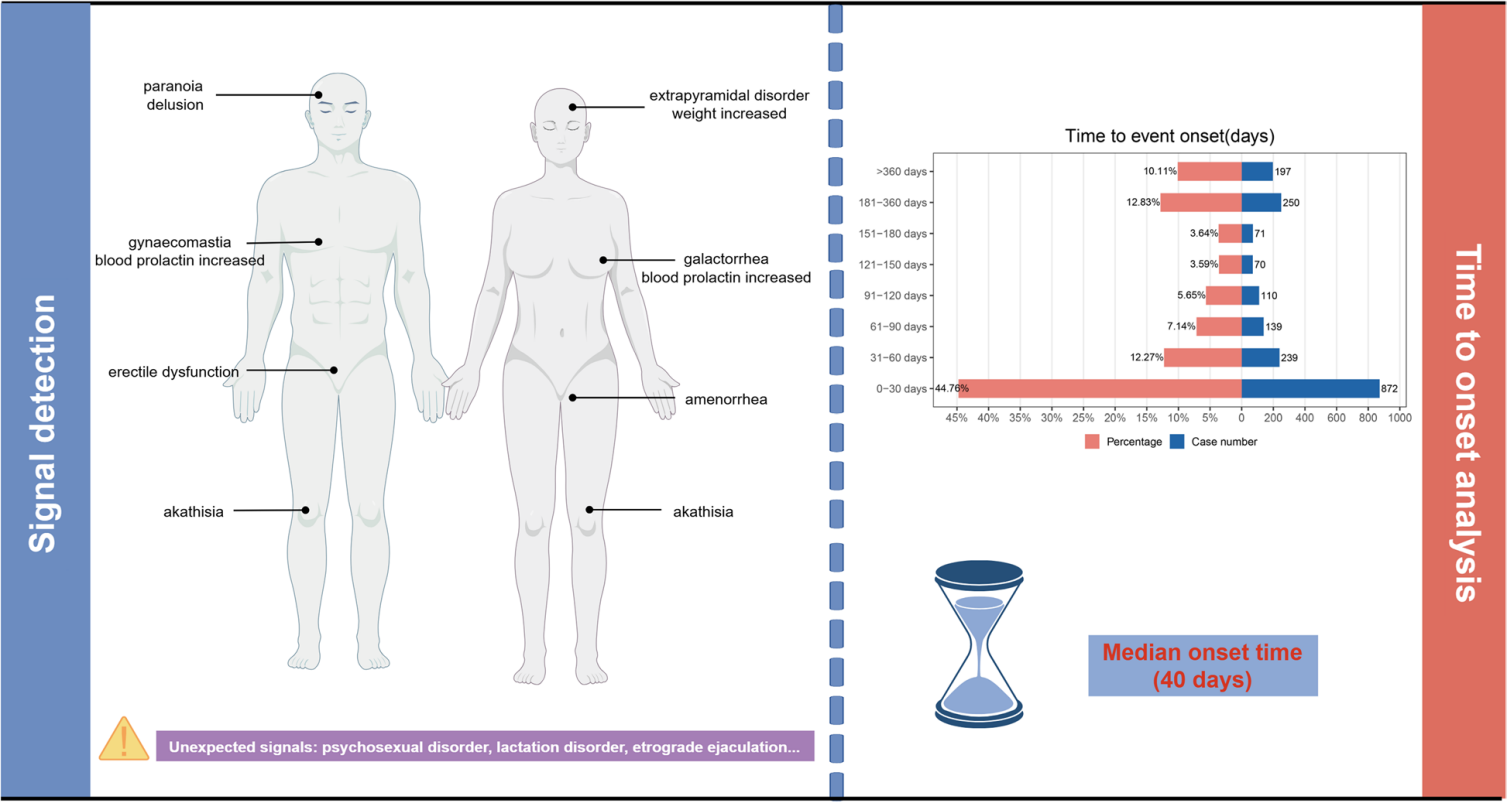
The image highlights the key findings from the FAERS database. In the detection of signal values stratified by gender, notable differences were observed between males and females, including unexpected signals such as psychosexual disorders. In the time to onset analysis, adverse drug events (ADEs) predominantly occurred within the first month of paliperidone palmitate dosing (44.76%), with a median onset time of 40 days

To the best of knowledge, this is the first study conducting a thorough and methodical pharmacovigilance analysis utilizing the FAERS and JADER databases to identify ADEs linked with paliperidone palmitate. The main findings in FAERS were presented in Fig. 6. Common signals observed in both genders include blood prolactin increased and akathisia. Additionally, females are more likely to experience weight increased, while males are more prone to paranoia and sexual dysfunction. Unexpected signals identified in the FAERS include psychosexual disorders, location disorders, and other adverse events. Most ADEs occur within the first month of drug administration (44.76%), with a median onset of 40 days. External validation using the JADER database identified 35 positive signals that overlapped with those found in FAERS, further supporting the FAERS results. Given the inherent limitations of the spontaneous reporting system databases, including potential biases and confounders, a prudent approach is warranted in interpreting our analysis results. Nonetheless, our research yields valuable insights into the safety profile of paliperidone palmitate. Moving forward, future prospective clinical trials or electronic health record data could be used in the future to better analyze and evaluate paliperidone palmitate-related ADEs.

## Supplementary Information

Supplementary Material 1. Fig S1: A bar chart displays the case number and frequency of the top 50 preferred terms (PTs) for paliperidone palmitate. SOC, System Organ Class.

Supplementary Material 2. Fig S2: The twenty-five most prominent ADE signals at the PT level, categorized by gender subgroups, are indicated. The arrows in Figures S2A and S2B denote instances where the lower boundary of the 95% CI of the ROR surpasses 25. C. Overlap of the top twenty-five signals in both subgroups. PRR, proportional reporting ratio; EBGM05, lower limit of 95% CI of EBGM; IC025, lower limit of 95% CI of the IC; ADE, adverse drug event; PT, preferred term; ROR, reporting odds ratio; CI, confidence interval.

Supplementary Material 3. Table S1: The signal intensity of ADE reports concerning paliperidone palmitate at the System Organ Class (SOC) level within the FAERS database is evaluated.

Supplementary Material 4. Table S2: 285positive signals satisfy the four disproportionality thresholds simultaneously in paliperidone palmitate are identified. PT entries are displayed in the descending order of case numbers. The asterisks indicate unexpected signals that are not listed in the drug label.

Supplementary Material 5. Table S3:Complete results of the sensitivity analysis. The asterisks indicate unexpected signals that are not listed in the drug label.

Supplementary Material 6. Table S4: Comprehensive gender-differentiated risk signal analysis results of paliperidone palmitate-associated adverse drug events (ADEs). the ROR algorithm's is not strictly a pharmacovigilance analysis method. Here, a means the number of reports of target ADE in female; b, number of reports of other ADEs in female; c, number of reports of target ADE in male; d, number of reports of other ADEs in male. Therefore, the ROR here is not a strictly defined ROR in pharmacoepidemiological perspective; we just use this algorithm for signal value calculation of gender-based signal strength differences.

Supplementary Material 7. Table S5: Detailed TTO analysis at the SOC levels in FAERS. TTO, time to onset; SOC, system organ class; Min, minimum; Max: maximum; IQR, interquartile range; q1, 1/4 quantile; q3, 3/4 quantile; SD, standard deviation; SE, standard error.

Supplementary Material 8. Table S6: Demographic baseline characteristics and comprehensive signal value calculations for paliperidone palmitate-associated adverse drug events (ADEs) from the JADER database. The overlap of positive signals identified from the FAERS and JADER databases is illustrated using a Venn diagram. JADER, Japanese Adverse Drug Event Report.

## Data Availability

Publicly available datasets were analyzed in this study. These data can be found at: https://fis.fda.gov/extensions/FPD-QDE-FAERS/FPD-QDE-FAERS.html and https://www.pmda.go.jp/index.html.
