# Supplementary figures and images for: A multidimensional assessment of adverse events associated with paliperidone palmitate: a real-world pharmacovigilance study using the FAERS and JADER databases
Source: BMC Psychiatry. 2025 Jan 20;25:52. doi: 10.1186/s12888-025-06493-0 (PMC11744949; doi:10.1186/s12888-025-06493-0)

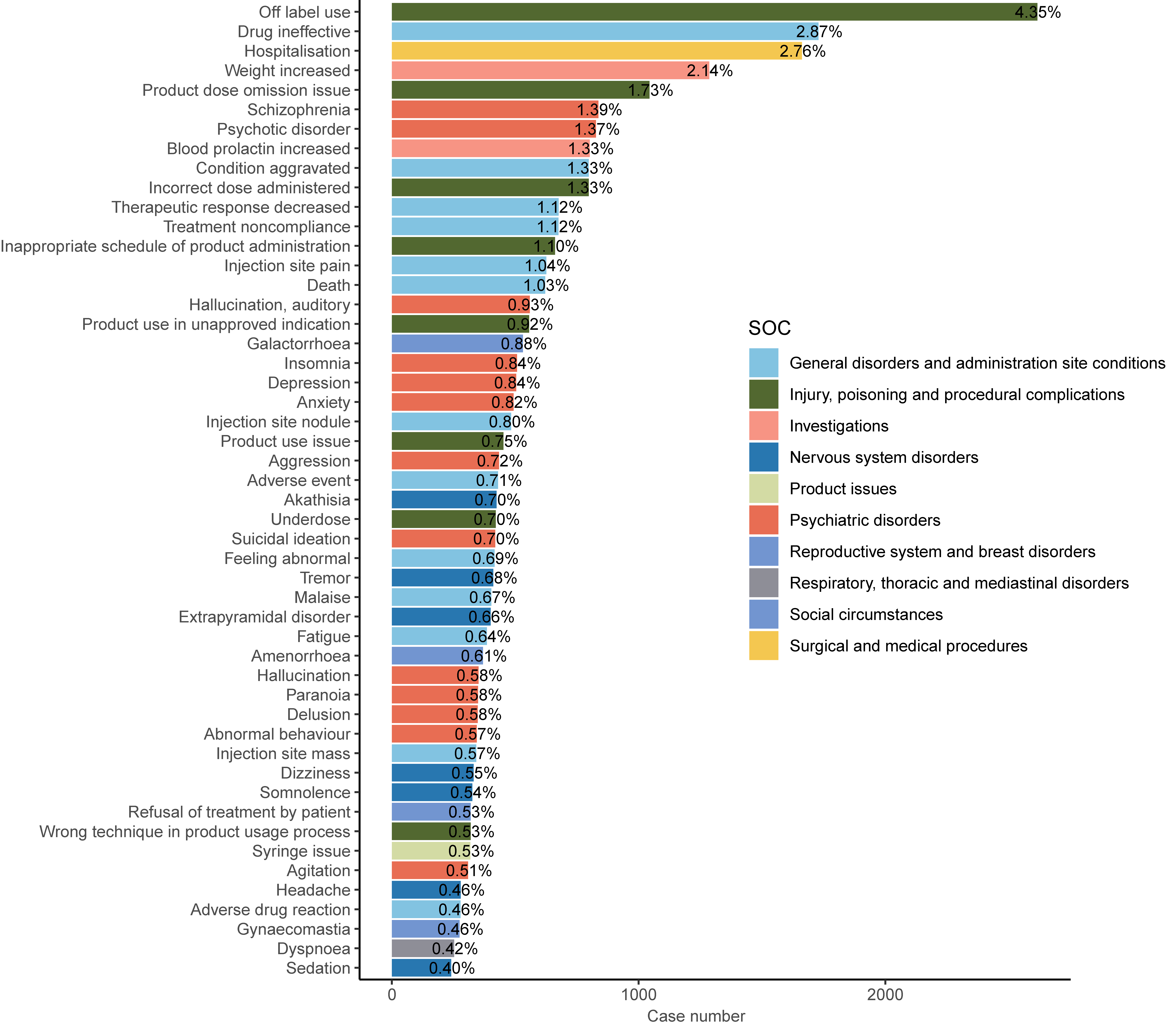

Supplement: Supplementary file 1 — Supplementary Material 1. Fig S1: A bar chart displays the case number and frequency of the top 50 preferred terms (PTs) for paliperidone palmitate. SOC, System Organ Class. [file 12888_2025_6493_MOESM1_ESM.tif]

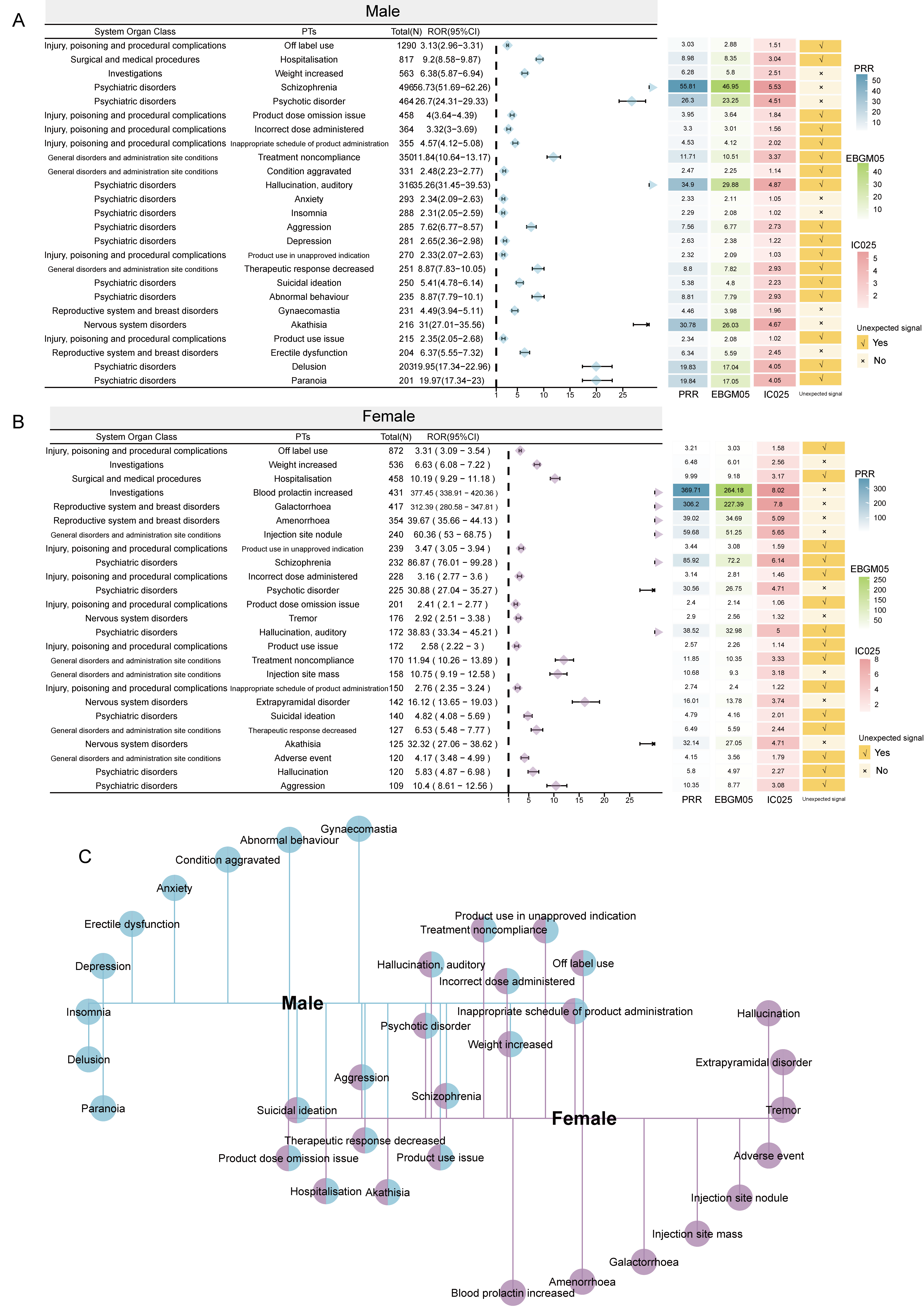

Supplement: Supplementary file 2 — Supplementary Material 2. Fig S2: The twenty-five most prominent ADE signals at the PT level, categorized by gender subgroups, are indicated. The arrows in Figures S2A and S2B denote instances where the lower boundary of the 95% CI of the ROR surpasses 25. C. Overlap of the top twenty-five signals in both subgroups. PRR, proportional reporting ratio; EBGM05, lower limit of 95% CI of EBGM; IC025, lower limit of 95% CI of the IC; ADE, adverse drug event; PT, preferred term; ROR, reporting odds ratio; CI, confidence interval. [file 12888_2025_6493_MOESM2_ESM.tif]
