# Supplementary material for: A multidimensional assessment of adverse events associated with paliperidone palmitate: a real-world pharmacovigilance study using the FAERS and JADER databases
Source: BMC Psychiatry. 2025 Jan 20;25:52. doi: 10.1186/s12888-025-06493-0 (PMC11744949; doi:10.1186/s12888-025-06493-0)

Demographic characteristics of ADEs reported in the JADER database (January 2013-June 2024) with paliperidone palmitate as the primary suspect drug. JADER, Japanese Adverse Drug Event Report.

| **Characteristics** | **Case number** | **Case proportion, %** |
| --- | --- | --- |
| **Gender, n (%)** | 1065 |  |
| Female | 456 | 42.8% |
| Male | 583 | 54.7% |
| Unknown | 26 | 2.4% |
| **Age**  <15 years | 13 | 1.2% |
| 15-65 years | 852 | 80.0% |
| >65 years | 91 | 8.6% |
| Unknown | 109 | 10.2% |
| **Weight**  <50 kg | 50 | 4.7% |
| 50-100 kg | 257 | 24.1% |
| >100 kg | 12 | 1.1% |
| Unknown | 746 | 70.1% |
| **Outcome** |  |  |
| Recovery (recovery but with sequelae) | 10 | 0.6% |
| Rehabilitation | 447 | 28.9% |
| Minor rehabilitation | 238 | 15.4% |
| Death | 315 | 20.3% |
| Non-rehabilitated | 121 | 7.8% |
| Missing | 418 | 27.0% |
| **Indication (top three)** |  |  |
| Schizophrenia | 2257 | 86.9% |
| Product use for unknown indications | 85 | 3.3% |
| Schizoaffective disorder | 13 | 0.5% |

The signal intensity of ADE reports concerning paliperidone palmitate at the System Organ Class (SOC) level within the JADER database is evaluated. JADER, Japanese Adverse Drug Event Report.

51 positive signals satisfy the four disproportionality thresholds simultaneously in paliperidone palmitate in JADER database are identified. PT entries are displayed in the descending order of case numbers. Signals not listed on the drug label are marked with asterisks.

Overlap of FAERS and JADER positive signals


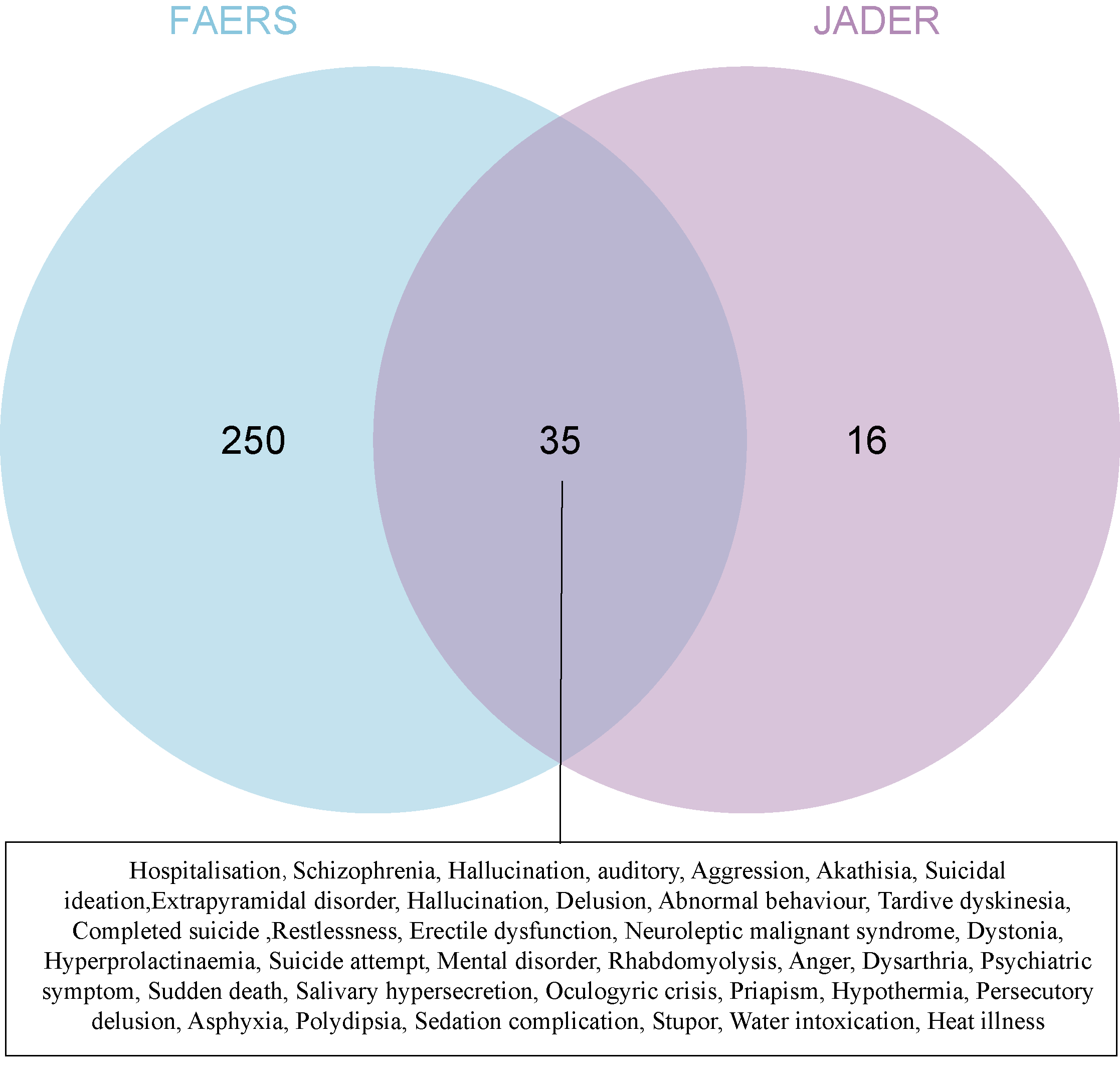

Supplement: Supplementary file 8 — Supplementary Material 8. Table S6: Demographic baseline characteristics and comprehensive signal value calculations for paliperidone palmitate-associated adverse drug events (ADEs) from the JADER database. The overlap of positive signals identified from the FAERS and JADER databases is illustrated using a Venn diagram. JADER, Japanese Adverse Drug Event Report. [file 12888_2025_6493_MOESM8_ESM.docx]
