# Supplementary material for: A multidimensional assessment of adverse events associated with paliperidone palmitate: a real-world pharmacovigilance study using the FAERS and JADER databases
Source: BMC Psychiatry. 2025 Jan 20;25:52. doi: 10.1186/s12888-025-06493-0 (PMC11744949; doi:10.1186/s12888-025-06493-0)
Supplement: Supplementary file 7 [file 12888_2025_6493_MOESM3_ESM.docx]

System Organ Class	SOC code	Case reports	ROR(95%CI)	PRR(χ2)	EBGM(EBGM05)	IC(IC025)
Psychiatric disorders	10037175	11690	4.06(3.98-4.14)	3.47(21636.32)	3.46(3.4)	1.79(1.76)
General disorders and administration site conditions	10018065	11582	1.1(1.08-1.12)	1.08(85.27)	1.08(1.06)	0.11(0.08)
Injury, poisoning and procedural complications	10022117	8967	1.6(1.56-1.63)	1.51(1704.21)	1.51(1.48)	0.59(0.56)
Nervous system disorders	10029205	6658	1.36(1.32-1.39)	1.32(560.74)	1.32(1.29)	0.4(0.36)
Investigations	10022891	3995	1.13(1.09-1.16)	1.12(52.66)	1.12(1.09)	0.16(0.11)
Reproductive system and breast disorders	10038604	2184	4.57(4.38-4.77)	4.44(5833.33)	4.42(4.26)	2.14(2.08)
Surgical and medical procedures	10042613	1916	2.35(2.24-2.46)	2.31(1433.38)	2.3(2.22)	1.2(1.14)
Musculoskeletal and connective tissue disorders	10028395	1598	0.48(0.46-0.51)	0.49(872.61)	0.49(0.47)	-1.02(-1.09)
Product issues	10077536	1541	1.52(1.44-1.6)	1.51(265.32)	1.5(1.44)	0.59(0.51)
Gastrointestinal disorders	10017947	1431	0.26(0.25-0.27)	0.28(2944.14)	0.28(0.27)	-1.85(-1.93)
Respiratory, thoracic and mediastinal disorders	10038738	1012	0.34(0.32-0.36)	0.35(1268.57)	0.35(0.33)	-1.51(-1.6)
Metabolism and nutrition disorders	10027433	1006	0.78(0.73-0.83)	0.78(61)	0.78(0.74)	-0.35(-0.44)
Infections and infestations	10021881	910	0.27(0.25-0.29)	0.28(1770.27)	0.28(0.27)	-1.83(-1.93)
Skin and subcutaneous tissue disorders	10040785	888	0.26(0.24-0.28)	0.27(1872.49)	0.27(0.25)	-1.9(-1.99)
Social circumstances	10041244	848	3.33(3.11-3.56)	3.3(1356.93)	3.29(3.11)	1.72(1.62)
Cardiac disorders	10007541	837	0.56(0.52-0.59)	0.56(293.17)	0.56(0.53)	-0.83(-0.93)
Eye disorders	10015919	653	0.53(0.49-0.57)	0.54(268.31)	0.54(0.5)	-0.9(-1.01)
Vascular disorders	10047065	595	0.46(0.43-0.5)	0.47(369.2)	0.47(0.44)	-1.1(-1.22)
Renal and urinary disorders	10038359	510	0.45(0.41-0.49)	0.45(341.53)	0.45(0.42)	-1.14(-1.27)
Neoplasms benign, malignant and unspecified (incl cysts and polyps)	10029104	274	0.16(0.14-0.18)	0.17(1188.24)	0.17(0.15)	-2.6(-2.77)
Endocrine disorders	10014698	264	1.71(1.52-1.93)	1.71(77.94)	1.71(1.54)	0.77(0.6)
Blood and lymphatic system disorders	10005329	262	0.26(0.23-0.29)	0.26(566.7)	0.26(0.23)	-1.95(-2.13)
Immune system disorders	10021428	207	0.3(0.26-0.34)	0.3(338.34)	0.3(0.27)	-1.73(-1.93)
Hepatobiliary disorders	10019805	143	0.27(0.23-0.31)	0.27(289.93)	0.27(0.23)	-1.9(-2.14)
Ear and labyrinth disorders	10013993	130	0.5(0.42-0.59)	0.5(65.88)	0.5(0.43)	-1(-1.26)
Pregnancy, puerperium and perinatal conditions	10036585	63	0.25(0.2-0.32)	0.25(140.86)	0.25(0.2)	-1.99(-2.35)
Congenital, familial and genetic disorders	10010331	37	0.2(0.15-0.28)	0.2(115.87)	0.2(0.16)	-2.3(-2.77)
